# Myeloperoxidase Antineutrophil Cytoplasmic Antibody-Associated Renal-Limited Vasculitis in a Young Adult Woman

**DOI:** 10.7759/cureus.21654

**Published:** 2022-01-27

**Authors:** Yukihiro Otaka, Daiki Uchida, Kinue Shimizu-Arii, Nobuyoshi Ishiyama, Keiko Kawai-Kowase

**Affiliations:** 1 General Internal Medicine, Hidaka-kai Hidaka Hospital, Gunma, JPN; 2 General Medicine, Gunma University Graduate School of Medicine, Gunma, JPN; 3 Pathology, Hidaka-kai Hidaka Hospital, Gunma, JPN

**Keywords:** renal-limited vasculitis, myeloperoxidase, microscopic polyangiitis, focal category, fibro-cellular crescent glomerulonephritis, antineutrophil cytoplasmic antibody

## Abstract

Antineutrophil cytoplasmic antibody (ANCA)-associated renal-limited vasculitis (RLV) is a minor subtype of small vessel vasculitis characterized by the inflammation of blood vessels, tissue damage, and loss of renal function localized in the kidney without systemic involvements. Here, we report a case of myeloperoxidase (MPO) ANCA-associated RLV in a young adult woman in Japan presenting chronic hematuria and newly overt proteinuria. Percutaneous renal biopsy revealed focal fibro-cellular crescent glomerulonephritis and the absence of other small vasculitides, tubular atrophy, and interstitial fibrosis. Therapeutic intravenous methylprednisolone pulse followed by oral prednisolone was administered as a remission induction. The patient’s serum MPO-ANCA level gradually decreased, coinciding with dramatic changes in proteinuria and hematuria after therapeutic glucocorticoid administration. Renal function was maintained within the normal range, and disease activity was well-tolerated throughout the follow-up period for more than 14 weeks. While the incidence of RLV is rare among younger patients, it occurs with asymptomatic hematuria and proteinuria, which is important in differentiating RLV from typical glomerulonephritis. The overall prognosis of ANCA-associated RLV potentially depends on the severity of extrarenal involvements. Early diagnosis, appropriate treatment, and regular maintenance are essential for controlling and treating RLV. Due to the nontypical case presented here, further investigation is recommended to improve the diagnosis strategies and treatment options for this disease.

## Introduction

Antineutrophil cytoplasmic antibody (ANCA)-associated vasculitis (AAV) is a rare disease characterized by the inflammation of blood vessels, endothelial injury, and damage of surrounding tissues [1]. Along with small vessel vasculitis, microscopic polyangiitis (MPA) features a loss of tolerance to neutrophil primary granule proteins, mainly myeloperoxidase (MPO). Instances of MPA typically occur in elderly populations and are accompanied by rapidly progressing glomerulonephritis (GN) with hematuria, proteinuria, and a progressive loss of renal function over a short period [2].

ANCA-associated renal-limited vasculitis (RLV) is a small vessel vasculitis localized in the kidney without systemic involvement [1]. ANCA-associated RLV shows many features that suggest it represents a renal-limited form of MPA. Additionally, ANCA-associated RLV showed relatively better outcomes compared with typical MPA or granulomatosis presenting with polyangiitis in terms of renal relapse, long-term dialysis, kidney transplantation, and mortality [3]. The mean age of RLV diagnosis was 60 years, with younger cases being uncommon [3-4]. Several studies of juvenile or young adult patients with AAV/ANCA-associated RLV have been reported [5]; however, more research is required to further characterize the long-term outcome of the disease.

## Case presentation

A 23-year-old Japanese woman with a three-year history of asymptomatic microscopic hematuria was referred to our department due to precipitating overt proteinuria and loss of appetite. The patient had a history of intermittent microscopic hematuria in her late childhood but was not referred to a doctor for further examination until recently. The patient’s microscopic hematuria had been documented at an annual medical checkup three years before admission, and she was referred to a urologist for further investigation. The patient displayed iron-deficiency anemia and chronic pruritus as comorbidities for the preceding six months, which had been treated with oral iron supplement and antihistamines. The patient was a nonsmoker and an occasional light drinker.

Upon admission, the patient’s body temperature was 37.3 °C, blood pressure was 150/98 mmHg, and pulse rate was 103 beats per minute. Height was measured at 163.0 cm, and body weight was 52.8 kg (body-mass index was 19.9). The patient had lost 3.6 kg during the preceding four months due to a prolonged loss of appetite. Other vital signs and physical findings were unremarkable, presenting no skin lesions or neurological disorders. Laboratory tests revealed a slight increase in white blood cell count but no evidence of systemic inflammation was seen (Table 1).

**Table 1:** Laboratory findings on admission Abbreviations: ANCA, anti-neutrophil cytoplasmic antibody; CH50, 50% hemolytic complement activity; dsDNA, double-stranded deoxyribonucleic acid; eGFR, estimated glomerular filtration rate calculated by the Japanese equation for Modification of Diet in Renal Disease [6]); FEIA, fluorescence enzyme immunoassay; GBM, glomerular basement membrane; Ig, immunoglobulin; LA, latex agglutination turbidimetric immunoassay; MPO, myeloperoxidase; PR3, proteinase 3; RBC, red blood cell; WBC, white blood cell

Items	Values	
WBC count	11,170	/μL
RBC count	4.64 × 10^6^	/μL
Reticulocyte	143,376	/μL
Hemoglobin	12.3	g/dL
Hematocrit	39.2	%
Platelet count	534,000	/μL
Total protein	7.2	g/dL
Albumin	4.4	g/dL
Lactate dehydrogenase	146	IU/L
Urea nitrogen	10.9	mg/dL
Creatinine	0.57	mg/dL
eGFR creatinine	108	mL/min/1.73m^2^
C-reactive protein	<0.05	mg/dL
Ferritin	106.3	ng/mL
Rheumatoid factor (LA)	3	IU/mL
IgA	127	mg/dL
IgE	113	IU/mL
IgG	1,325	mg/dL
IgM	133	mg/dL
CH50	57	U/mL
Complement component 3	99	mg/dL
Complement component 4	15.0	mg/dL
Anti-nuclear antibody	Less than 1:40	
MPO-ANCA (FEIA)	681.0	IU/mL
PR3-ANCA (FEIA)	<0.5	IU/mL
Anti-GBM antibody (FEIA)	0.6	U/mL
Cryoglobulin	Negative	
Anti-dsDNA antibody (FEIA)	1.1	IU/mL
Hepatitis-B surface antigen	Negative	
Hepatitis-C virus antibody	Negative	

Progressive proteinuria (908.8 mg per day) and microscopic hematuria (50-99 of red blood cells per high power field) were observed; however, subsequent deformation of urinary red blood cells and casts were not detected. Renal function was preserved (24-hour creatinine clearance, 160 mL/min), and the iron-deficiency anemia was well-controlled with medication. Serum MPO-ANCA presented a high titer of 681.0 IU/mL well above the normal range (<3.5 IU/mL). There was no evidence of lung consolidation, interstitial changes, or honeycomb signs on her chest X-ray and computed tomography.

**Figure 1:**
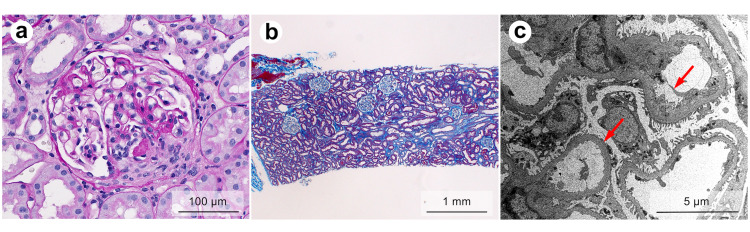
Histopathology of renal biopsy specimens (a) Focal fibro-cellular crescent formation (periodic acid–Schiff stain); (b) Unremarkable tubular atrophy and interstitial fibrosis (Masson’s trichrome stain); (c) Focal widening of the subendothelial spaces in the glomerulocapillary (arrows), lamination, and uneven thickening and thinning of basement membrane (electron microscopy)

Percutaneous renal biopsy specimens contained 40 glomeruli, including three adhesive lesions, three cases of segmental sclerosis, and one fibrocellular crescent (Figure 1a).

The rupture of the glomerular capillary wall, fibrin precipitation, and fibrin thrombus formation was observed in some glomeruli, showing focal necrotizing GN. There were no inflammatory changes, no double contour or spike formation of the basement membrane, and no enlargement of the mesangial region. Intratubular erythrocyte casts were observed, but there was no atrophy of renal tubules, and interstitial inflammation or fibrosis was unremarkable (Figure 1b). No evidence of vascular sclerosis or vasculitis was found in the interlobular arteries, venules, or arterioles. A small deposit of immunoglobulin M in the mesangial region was observed, but otherwise, few deposits were seen via immunofluorescent staining. Electron microscopy did reveal focal glomerular collapse, widening on the subendothelial spaces, lamination, and thickening and wrinkling of the basement membrane, representing the effects of vasculitis (Figure 1c). Electron dense deposits were not observed.

**Figure 2:**
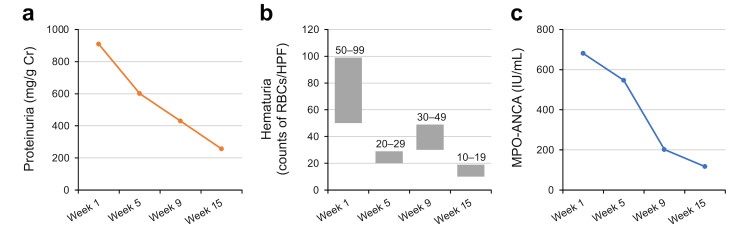
The changes in proteinuria, microscopic hematuria, and serum myeloperoxidase-antineutrophil cytoplasmic antibody (MPO-ANCA) throughout treatments

Based on the patient’s clinical and pathological findings, the patient was diagnosed as MPO-ANCA-associated RLV, with a high titer of circulating MPO-ANCA and pauci-immune necrotizing GN. The Initial Birmingham Vasculitis Activity Score-2003 (BVAS-2003) at diagnosis was 11, accorded by newly diagnosed hypertension (+4), persistent weight loss (+2), proteinuria (+2), and hematuria (+3). The remission induction therapy comprised intravenous methylprednisolone pulse (1 g per day) followed by oral prednisolone (50 mg daily for 2 weeks, then tapered gradually). Although proteinuria and hematuria were dramatically reduced after glucocorticoid administration (Figures 2a-2b), serum MPO-ANCA remained well above the normal limit; however, the titer progressively decreased along with maintenance therapy (Figure 2c).

During the follow-up period over the next 14 weeks, the estimated glomerular filtration rate was maintained at the normal range between 90 and 110 mL/min/1.73 m^2^, and the reassessed BVAS-2003 was 5, accorded by the still persistent proteinuria and microscopic hematuria at week 15.

## Discussion

AAV is a rare disease characterized by microvascular endothelial inflammation leading to extravascular inflammation, progressive injury, tissue damage, fibrosis, and loss of function. Among the small vessel vasculitis defined by the 2012 revised Chapel Hill Consensus Conference [1], MPA features a loss of tolerance to neutrophil primary granule proteins, which predominate MPO in 60% of cases. RLV, a minor subtype of single-organ AAVs, is characterized by small vessel vasculitis localized in the kidney occurring in the absence of systemic involvement [1]. Approximately 80% of patients with RLV will be positive for ANCA, predominantly MPO-ANCA, which has many features to suggest that it represents a renal-limited form of MPA [3]. The incidence of MPA was 0.5-24.0 cases per one million person-years, and the typical age of disease onset is reported at 55-75 years [2]. Similarly, the mean age at diagnosis of RLV was 59.8 years [3-4]. The sex ratio is estimated to be roughly 1:1, and geographically, MPA predominates in Asian regions such as Japan and China.

In a single-center study for rapidly progressive GN, pauci-immune crescentic GN, including MPA and RLV, was observed in 42% of younger patients aged between one and 20 years [7]. Moreover, unlike in adults, AAV in children is likely to be more predominant among women rather than men [8-9]. This case showed a previously healthy young adult woman with a final diagnosis of MPO-ANCA-associated RLV. Although the incidence of RLV at a young age is extremely rare, it may occur with asymptomatic hematuria and overt proteinuria, the comorbidity of which is important as one of the differentials from other typical GNs such as immunoglobulin A nephropathy.

Among the four patterns of glomerular lesions defined by a histopathological classification in AAV, the focal category (≥50% normal glomeruli regardless of tubulointerstitial lesions or renal function), which was seen in this case, showed the best prognosis, i.e., relatively preserved renal function and favorable renal outcome, compared with the other types of injuries [10]. More recent studies have shown that a renal risk score using the percentages of normal glomeruli, tubular atrophy/interstitial fibrosis, and estimated glomerular filtration rate at the time of diagnosis will predict renal survival and the risk of end-stage renal disease (ESRD) [11]. Among these parameters, the percentage of normal glomeruli was the strongest independent predictor of mortality from ESRD [11].

## Conclusions

In summary, the prognosis of MPA or ANCA-associated RLV potentially depends on the severity of organ involvement. Early diagnosis, appropriate treatment, and constant maintenance are essential for all patients, including adolescents and children. Further investigation is required to improve the strategies of diagnosis and treatment options for this disease, as its occurrence in younger patients is poorly understood.
